# Is the mothers’ country of birth associated with the sex of their offspring in England and Wales from 2007 to 2011?

**DOI:** 10.1186/1471-2393-14-332

**Published:** 2014-09-25

**Authors:** Cameron Smith, Andrew Fogarty

**Affiliations:** Medical Student, University of Nottingham, 88: Harrington Drive, Lenton, Nottingham, NG7 1JN UK; Reader in Clinical Epidemiology, Division of Epidemiology & Public Health, University of Nottingham, Clinical Sciences Building, Nottingham City Hospital, Hucknall Road, Nottingham, NG5 1PB UK

**Keywords:** Sex ratio, India, England and Wales, Africa

## Abstract

**Background:**

Preference for sons in India has resulted in a skewed sex ratio at live birth, probably as a consequence of female feticide. However, it is unclear if these cultural preferences are also currently present in communities who have emigrated from India to England and Wales.

**Methods:**

Data of all live births in England and Wales from 2007–2011 were obtained from the Office of National Statistics. A logistic regression analysis was used to compare the probability of having a male infant in mothers born inside the United Kingdom (UK) to those born outside the UK, stratified by mothers’ region and country of birth.

**Results:**

Mothers born in India were not observed to be giving birth to disproportionately more boys than mothers that were born in the UK (Odds Ratio OR: 1.00, 95% Confidence Interval CI: 0.98 - 1.02), although an excess of male births were observed in mothers born in South-East Asia (OR 1.03; 95% CI: 1.01-1.05, p = 0.005), the Middle East (OR 1.02; 95% CI: 1.00-1.05, p = 0.047), and South America (1.04; 95% CI: 1.00-1.07, p = 0.025). Mothers who were born in Africa were found to be less likely to give birth to boys than girls when compared to mothers born in the UK (OR: 0.98, 95% CI: 0.97–0.99), and this observation was attributable to women born in East and West Africa.

**Conclusion:**

There was no evidence of an excess of males born to women from India in England and Wales. An excess of males were observed in mothers born in South-East Asia, the Middle East and South America. Women born in Africa are less likely to give birth to boys than UK born mothers, an observation that is consistent with previous data.

## Background

The worldwide sex ratio at live birth (defined as the number of males born divided by the number of females) is approximately 1.05 [1]. The sex ratio has been decreasing over recent decades in several industrial countries [2] including the United Kingdom [3]. However, in India and other Asian countries, the sex ratio is elevated [4, 5], and this is thought to be a consequence of gender selection, due to the cultural and economic importance placed on male offspring, who are more likely to earn a higher wage, continue the family line and provide assistance to their parents in old age [6]. Studies from Norway [7] and the USA [8] have reported higher than expected male:female sex ratios at live birth in women born in the Indian subcontinent, consistent with the practice of female feticide. Data from England and Wales also reported an increase in the proportion of males born to Indian mothers from 1969 to 2005, and considered that this was consistent with the hypothesis that sex-selective abortion was practiced within this ethnic group [9].

We have used contemporary national statistical data from England and Wales to test the hypothesis that women born in India would be more likely to have male offspring during the period from 2007 to 2011. As this has coincided with a period when many individuals from many countries are arriving in England and having children, we also had the opportunity to assess the probability of having a male infant in women from different regions of the world compared to those born in the United Kingdom.

## Methods

### Study population

Data was obtained on all live births in England and Wales from 2007–2011 from the Office of National Statistics. Mothers are asked to provide their country of birth when they register the birth of their child (within 42 days of the child being born). The data were provided summarised by region for most individuals except for those who were born in India, Pakistan, Bangladesh and Poland where data were available at the country level.

### Variables of interest

The main outcome variable for this study was the probability of having a male infant compared to a female. The exposure variable was the country or region of the mother’s birth.

### Data analyses

We calculated the overall sex ratio for each of the areas by dividing the total male births by the total female births. The primary analysis used logistic regression to generate odds ratios for having a male child for mothers born in the geographic area of interest compared to mothers who were born in the UK for all five years of available data (2007–2011). STATA version 12.1 was used to carry out all data analyses (StataCorp, Texas, USA). The analysis and manuscript preparation were in accordance with STROBE guidelines.

## Results

Data were available for 3 552 050 births from 2007 to 2011, increasing from 690 013 births in 2007 to 723 913 births in 2011. Of these, 2 680 239 (75%) were to mothers born in England and Wales and 871 705 (25%) were to mothers who were not born in England and Wales. Data on the maternal country of birth were not available for 106 (<0.01%) births.

### Continental analysis

In the analysis stratified by continent, mothers who were born in Africa were less likely to have a male child than those who were born in the UK, with an odds ratio of 0.98 (95% confidence intervals: 0.97 to 0.99). Mothers who were born in Europe (excluding UK), the Americas and the Carribean, the Middle East and Asia and Antarctica and Oceania had the same probability of having a male child as mothers born in the UK (Table 1).

**Table 1:** **The association of place of mother**’**s birth for sex ratio at live birth for all births in England and Wales 2007**-**2011**

Region of mother’s birth	Number of males born	Number of females born	Sex Ratio	Odds ratio*	95% confidence interval	p value
UK	1,375,306	1,304,933	1.054	1	-	-
Outside UK	446,166	425,539	1.048	0.995	0.99 – 1.00	0.035
**Outside UK divided by continent**						
Europe	140 445	133254	1.054	1.00	0.99 - 1.01	0.992
Africa	99 771	96 422	1.035	0.98	0.97 - 0.99	<0.001
The Americas and the Caribbean	29 145	27 435	1.062	1.01	0.99 - 1.02	0.350
Middle East and Asia	167 146	159 299	1.049	1.00	0.99 - 1.00	0.231
Antarctica and Oceania	9 659	9 129	1.058	1.00	0.98 - 1.03	0.790

*The reference group is mothers who were born in the UK.

### Regional analysis

Mothers who were born in East Africa and West Africa had lower probabilities of having a male child than those who were born in the UK with odds ratios (OR) of 0.97 (95% CI: 0.96-0.99) and 0.97 (95% CI: 0.96-0.99) respectively (Table 2). Women who were born in the Middle East (OR 1.02; 95% CI: 1.00-1.05), South-East Asia (OR 1.03; 95% CI: 1.01-1.05) and South America (OR 1.04; 95% CI: 1.00-1.07) had a higher probability of having a male child than women born in the UK.

**Table 2:** **The association between geographical region of mothers**’ **birth and sex ratio at live birth in England and Wales 2007**-**2011**

Region of mother’s birth	Number of males born	Number of females born	Sex ratio	Odds ratio*	95% Confidence interval	p value
UK	1 375 306	1 304 933	1.054	1	-	-
EU	121 411	115 543	1.051	0.997	0.99 - 1.01	0.486
Rest of Europe (non European Union)	19 034	17 711	1.075	1.020	1.00 - 1.04	0.063
North Africa	9 258	8 755	1.057	1.00	0.97 - 1.03	0.823
Western Africa	32 674	31 869	1.025	0.97	0.96 - 0.99	0.001
Central Africa	6 688	6 375	1.049	1.00	0.96 – 1.03	0.79
Eastern Africa	38 822	37 875	1.025	0.97	0.96 - 0.99	<0.001
Southern Africa	12 101	11 313	1.070	1.01	0.99 - 1.04	0.259
North America	11 356	10 783	1.053	1.00	0.97 - 1.03	0.956
Central America	828	842	0.983	0.93	0.85 - 1.03	0.157
South America	8 455	7 744	1.092	1.04	1.00 - 1.07	0.025
Caribbean	8 506	8 066	1.055	1.00	0.97 - 1.03	0.970
Middle East	15 936	14 780	1.078	1.02	1.00 - 1.05	0.047
Central Asia	793	751	1.056	1.00	0.91 - 1.11	0.970
Eastern Asia	14 244	13 437	1.060	1.01	0.98 - 1.03	0.631
Southern Asia	118 432	113 979	1.039	0.99	0.98 - 0.99	0.001
South East Asia	17 522	16 126	1.087	1.03	1.01 - 1.05	0.005
Australasia	9 057	8 532	1.062	1.01	0.98 - 1.04	0.635
Other Oceania	601	595	1.010	0.96	0.86 - 1.07	0.463

*The reference group is mothers who were born in the UK.

### Country analysis

Mothers who were born in India had no increased probability of having a male child compared to mothers who were born in the UK with an odds ratio of 1.00 (95% CI: 0.98-1.02). Women who were born in Pakistan and Bangladesh had a lower probability of having a male child than women who were born in the UK with odds ratios of 0.98 (95% CI: 0.97-1.00) and 0.98 (95% CI: 0.96-1.00) respectively (Table 3).

**Table 3:** **Births by individual country 2007**-**2011**

Country	Number of males born	Number of females born	Sex ratio	Odds ratio*	95% Confidence Interval	p value
UK	1,375,306	1,304,933	1.054	1	-	-
Poland	44235	42216	1.048	0.99	0.98 - 1.01	0.435
India	33565	31866	1.053	1.00	0.98 - 1.02	0.942
Pakistan	46404	44738	1.037	0.98	0.97 – 1.00	0.018
Bangladesh	21630	20957	1.032	0.98	0.96 – 1.00	0.032

*The reference group is mothers who were born in the UK.

## Discussion

Our analysis of national statistical data from England and Wales over a five year period has demonstrated a number of interesting observations. Firstly, there is no evidence that mothers born in India have an excess of male offspring, contrary to our hypothesis. Secondly, mothers from other regions of the world such as the Middle East, South-East Asia and South America have more male infants than those in the reference group of mothers who were born in the United Kingdom. Finally, women who were born in West and East Africa were less likely to have male offspring than the reference group.

The strength of this dataset is that it is nationally representative of England and Wales, with data collected on the mother’s country of origin as part of the data collection at the registration of the birth. This is seen in the fact that the mother’s country of birth was missing in less than 0.01% of births registered during the study period. Another strength is that over this time period, England and Wales have experienced relatively high levels of immigration from a wide number of regions of the world. This provides a natural experiment to investigate any potential differences in sex ratio at birth in mothers from differing areas of the world.

However, we also need to consider the limitations of this analysis as these are important in interpreting the results. We only have data on the mothers country of birth, and not that of the age of the mother’s arrival in England and Wales, nor the age of the father which obviously is an important factor in sex determination [10]. The reference group is mothers born in England and Wales and while this has a sex ratio of 1.054 which is within the expected range [1], this will itself be a heterogenous group of individuals. Finally, we have necessarily tested a number of hypotheses so we are unable to discount the possibility that some of our significant statistical associations are spurious.

The sex ratio of males to females born to Indian-born mothers in England and Wales steadily increased from 1969 to 2005, with the most rapid change occurring in the 1990s [9]. In this analysis, the authors reported sex ratios at birth for mothers born in India in the early 21^st^ century ranging from approximately 1.07 to 1.11, while our more recent data from 2007 to 2011 gave a comparable value of 1.053. We used similar data to that which contributed to the prior analysis and did not observe a significant increase in the probability of mothers born in India having male offspring compared to mothers born in the UK. This is consistent with the interpretation that if the practice of sex-selective abortion was present in the last decades of the 20^th^ century, it is less common nowadays. One potential explanation for this change is that the nature of immigrants who leave India for England and Wales has changed [11], and those who arrive in the 21^st^ century are less susceptible to the cultural and economic pressures that create the environment where sex-selective abortion is acceptable. A recent analysis of data from the Norwegian Central Population Register similarly did not demonstrate an excess of male births compared to females from 2006 to 2012 [12].

Our analysis also identified mothers from countries in South East Asia, the Middle East and South America as having higher odds ratios for having boys than mothers born in the UK. Previous studies have suggested that there has been a rapid increase in the number of males being born in Vietnam [13], and also that Filipino immigrants to the USA have a higher than expected sex ratio [8]. Some counties in the Middle East have a gender bias in mortality in females, and this has been interpreted as a possible consequence of the discriminatory treatment of women [14]. Where it exists, such prejudice may conceivably begin after conception. However, our data are observational and do not allow firm conclusions to be drawn as to the explanation of these differences. Despite the ability to radiologically identify the sex of the fetus soon after the end of the first trimester, sex-specific abortion is illegal in the UK.

Our data also showed that mothers from East and West Africa had a lower odds ratio of giving birth to boys than mothers from the UK that was unlikely to have occurred by chance. This observation was first reported in 1984 using data from both England and Wales [15] and the USA [16], and corroborated in subsequent studies [1–9, 17]. One potential explanation for this observation is that tropical latitude may be in itself an important factor in determining the sex ratio at live birth, with humans living in more tropical areas having an increased chance of having female offspring compared to those who do not [18]. Other possibilities include differential exposures to parasite infection [19] or other environmental factors that may also modify national mortality rates [20].

## Conclusions

In summary, our data demonstrate no evidence that mothers born in India have an excess of male births when compared to mothers born in the UK, although mothers born in the Middle East, South East Asia and South America do have higher odds ratios for giving birth to boys than mothers from the UK. We also observed that mothers from Africa are giving birth to fewer boys compared to mothers born in the UK. Our data do not allow distinctions between biological explanations and cultural practices, but are hypothesis generating and have the potential to be tested in other countries with relatively high levels of global immigration.

### Ethical approval

No ethical approval was required as fully anonymised data was used; therefore no ethics committee was consulted before proceeding with this project.

## Abbreviations

UK: United Kingdom
OR: Odds ratio
95% CI: 95% Confidence interval
USA: United States of America.
